# Emergency Medical Service Use Among Latinos Aged 50 and Older in California Counties, Except Los Angeles, During the Early COVID-19 Pandemic Period

**DOI:** 10.3389/fpubh.2021.660289

**Published:** 2021-08-23

**Authors:** Esmeralda Melgoza, Hiram Beltrán-Sánchez, Arturo Vargas Bustamante

**Affiliations:** ^1^Department of Community Health Sciences, UCLA Fielding School of Public Health, Los Angeles, CA, United States; ^2^UCLA Latino Policy and Politics Initiative, Los Angeles, CA, United States; ^3^UCLA California Center for Population Research, Los Angeles, CA, United States; ^4^Department of Health Policy and Management, UCLA Fielding School of Public Health, Los Angeles, CA, United States

**Keywords:** middle aged and older adults, Latinos, emergency medical services, COVID-19, health disparities, California (USA)

## Abstract

The COVID-19 pandemic has disproportionately affected Latino adults aged 50 and older in California. Among adults aged 50–64, Latinos constitute approximately one-third (32%) of the population, but over half (52%) of COVID-19 cases, and more than two-thirds (64%) of COVID-related deaths as of June 2, 2021. These health disparities are also prevalent among Latinos 65 years and older who constitute 22% of the population, but 40% of confirmed COVID-19 cases and 50% of COVID-related deaths. Emergency medical services (EMS) are an essential component of the United States healthcare system and a vital sector in COVID-19 response efforts. Using data from the California Emergency Medical Services Information System (CEMSIS), this study examines racial and ethnic differences in respiratory distress related EMS calls among adults aged 50 and older in all counties except Los Angeles. This study compares the early pandemic period, January to June 2020, to the same time period in 2019. Between January and June 2019, Latinos aged 50 and older had statistically significantly lower odds of respiratory distress related EMS calls compared to Blacks, Asians, and Whites. During the early pandemic period, January to June 2020, Latinos aged 50 and older had statistically significantly lower odds of respiratory distress related EMS calls compared to Blacks but slightly higher odds compared to Whites. Differences by race/ethnicity and region were statistically significant. Understanding EMS health disparities is crucial to inform policies that create a more equitable prehospital care system for the heterogeneous population of middle aged and older adults.

## Introduction

The novel coronavirus disease 2019 (COVID-19) pandemic has impacted the health and well-being of individuals across the world. As of June 2, 2021, approximately 33.3 million confirmed COVID-19 cases and 595,000 COVID-related deaths have been reported in the United States (U.S.) (1). In California, the most populous state in the U.S., approximately 3.6 million confirmed COVID-19 cases and 62,000 COVID-related deaths have been reported as of June 2, 2021 (1). Although the impact of the COVID-19 pandemic has been generalized, infections and deaths have disproportionately affected racial and ethnic minority populations, including Latinos (2, 3).

The health disparities affecting the Latino population have been particularly alarming in California (4). COVID-related health disparities among the Latino population are present in all age groups but are particularly high among individuals aged 50 and older (5). Among adults aged 50–64, Latinos constitute 32% of the state's population, but 52% of COVID-19 cases and 64% of COVID-related deaths as of June 2, 2021 (5). These health disparities are also prevalent among Latinos 65 years and older who constitute 22% of the state's population, but 40% of confirmed COVID-19 cases and 50% of COVID-related deaths as of June 2, 2021 (5). The health disparities exposed by the COVID-19 pandemic require immediate public health action and policy change to address the needs of disproportionately impacted populations, including middle aged and older adults who are Latino (6).

Health disparities in the Latino population are influenced by several factors, including racism, poverty, lack of or inadequate health insurance, job exposure, overcrowded housing, and higher prevalence of pre-existing medical conditions such as diabetes, cardiovascular disease, and hypertension (2, 7–9). Likewise, the overwhelming impact of COVID-19 on Latinos aged 50 and older is attributed to multiple factors stemming from structural racism, including early health deterioration and an accumulation of disadvantage over time (2). Latinos are also more likely to engage in low-paying, essential work, and use public transportation compared to Whites (8). Eligibility and access to health insurance among the Latino population is also influenced by immigration status, English language proficiency, and place of birth (10, 11). Uninsured and underinsured individuals are less likely to have a regular source of health care and are more likely to delay seeking health care services, which may lead to negative health consequences (10, 12). All of these factors increase the risk of COVID-19 infection and death among Latinos, especially individuals aged 50 and older (8).

Emergency medical services (EMS) are an essential component of the healthcare system in the U.S. and are a vital sector in COVID-19 response efforts. The activation of the EMS system occurs after a 9-1-1 call initiates a series of events, such as dispatch and arrival of personnel and resources, on-scene care, and ambulance transports. The EMS system is also characterized by the provision of healthcare services in the prehospital care setting. Previous studies show that Latino adults aged 50 and older are less likely to use EMS compared to other racial and ethnic groups (13–15). The disparities in EMS use by race and ethnicity are best documented in the cardiac arrest and stroke literature (16, 17). Latinos are less likely to call 9-1-1 after the onset of cardiac arrest and stroke symptoms, which are associated with a worse prognosis for the patient (16, 17). Latinos are also less likely to use an ambulance as the primary mode of transportation to the emergency department (ED) compared to Whites (18). Documented barriers to emergency service use among Latinos include general distrust in calling 9-1-1, financial concerns, immigration status, and patient-provider language discordance (19–22). Latino emergency medical technicians (EMTs) and paramedics are also underrepresented in the EMS workforce, which may contribute to the general distrust in emergency services and patient-provider language discordance (20, 23, 24).

During the early pandemic period, the overall use of emergency services in the U.S. rapidly declined (25, 26). Starting the first week of March 2020, the U.S. White House declared COVID-19 a national emergency (27). This same week EMS use decreased by 26% (25). Emergency department visits also decreased by 42% during the early pandemic period compared to the same time period during the previous year (26). Changes in the types of EMS calls also characterized the early pandemic period (25). Injury-related EMS calls decreased from 18 to 15% (25). The decline in injury-related EMS calls was at least partially attributed to behavioral changes that resulted from stay-at-home orders, such as less participation in risky activities, including driving (25). On the contrary, on-scene deaths doubled from 1.5 to 3% (25). The increase in on-scene deaths was partially attributed to fear of COVID-19 infection, which contributed to decreases in the use of ambulatory and emergency health care services (25, 28).

Previous studies show the impact of the COVID-19 pandemic on the overall use and types of EMS calls received during the early pandemic period (25, 29). The current study investigates racial and ethnic disparities in respiratory distress related EMS calls among adults aged 50 and older during the early pandemic period, January to June 2020, compared to the same time period in 2019. Respiratory distress related EMS calls are likely to increase during the period of study as these symptoms are common in patients with COVID-19. The focus of this study is on respiratory distress related EMS calls among Latino adults aged 50 and older since this population has been overrepresented in the number of COVID-19 cases and deaths in California.

## Methods

### Data Source

The California Emergency Medical Services Information System (CEMSIS) is the first state-wide demonstration project in California that offers a secure and centralized repository of EMS data. CEMSIS is overseen by the California Emergency Services Authority (EMSA), which is charged with developing, implementing, and evaluating EMS systems in the state. California Emergency Medical Services Information System collects EMS data according to the National Emergency Medical Services Information System (NEMSIS) version 3.4 data standards (30). The NEMSIS data dictionary is used when coding CEMSIS data, although not all variables in the national database are available in the state database (30).

California Emergency Medical Services Information System is an administrative database that is used for multiple purposes in addition to research, including studying local variation in data quality and local capacity for health information exchange. Currently, 32 out of 33 local emergency medical service agencies (LEMSAs) submit EMS data to CEMSIS. Los Angeles County is the only LEMSA not currently submitting EMS data to CEMSIS because it is in the testing phase. The testing phase refers to the process of examining data exchange software, programs, both technical capabilities and compatibilities with that of CEMSIS, and ensuring that the data sharing process is compliant with the NEMSIS.

California Emergency Medical Services Information System consists of electronic patient care reports (ePCRs) that are completed by each responding unit. As a result, several ePCRs may be completed per incident. The dataset requested from EMSA excludes duplicates or multiple ePCRs per EMS incident. As a result of the health information contained in CEMSIS, all data requests require approval from EMSA. After approval, the dataset is deidentified and sent to the research team in a password protected file.

### Study Design

This study is a 2-year comparative retrospective cross sectional analysis of CEMSIS data from January to June 2019 and January to June 2020. California Emergency Medical Services Information System is the only free and publicly available EMS dataset that includes almost all of the LEMSAs in California. California has a total of 33 LEMSAs. Every LEMSA submits EMS data to CEMSIS with the exception of Los Angeles County. Thus, 32 out of 33 LEMSAs submit EMS data to CEMSIS. The study does not include data from 2017 and 2018 because of the limited number of LEMSAs submitting data during these years: only 23 out of 33 in 2017 and 31 out of 33 in 2018. This study only includes data on EMS calls with patient contact. In certain cases, EMS providers do not find the patients on-scene. These types of calls are not included in the study because sociodemographic and health outcome data on these patients are not collected.

### Data Analysis

For this study, the dataset is restricted to EMS incidents with patient contact for the months of January to June 2019 and January to June 2020. The examined variables include patient age, gender, race and ethnicity, home regions, and provider primary impressions. The data are restricted to adults aged 50 and older. Age is coded as a continuous variable, although the results for age as a categorical variable are included in Tables A1, A2 in the Appendix. Gender is a binary variable that is coded as 1 for women and 2 for men. The reference category is men. California Emergency Medical Services Information System includes over 100 different categories for race and ethnicity. In this study, a patient's race and ethnicity is coded as a five-category variable−0 for Other, 1 for Latino, 2 for Black, 3 for Asian, and 4 for White. The reference category for the race and ethnicity variable is Latino since we are primarily interested in comparing the presence or absence of respiratory distress related EMS calls between Latinos and other racial and ethnic groups. Patient home countries are aggregated to create home regions, which are larger geographic units composed of multiple counties. Patient home region is coded as a five-category variable: Other Region is coded as 0, Northern California is coded as 1, Bay Area is coded as 2, Central California is coded as 3, and Southern California is coded as 4. The reference category is Southern California since it is the region with the largest population overall. For the purposes of this study, the dependent variable is binary—presence or absence of respiratory distress. The dependent variable is coded as 0 for a non-respiratory distress related EMS call and 1 for a respiratory distress related EMS call.

The main independent variable of interest is patient race and ethnicity, with a focus on Latinos in comparison to other racial and ethnic groups. Standard univariate analysis is used to characterize the sample of EMS calls among adults aged 50 and older. Bivariate analysis is used to determine the association between predictor variables, age, gender, race and ethnicity, home region, and the presence or absence of respiratory symptoms. Binary logistic regression models are constructed to identify and measure the independent associations between predictors, including age, gender, race and ethnicity, home region, and the binary outcome variable, presence or absence of respiratory symptoms. Each time period under study, January to June 2019 and January to June 2020 uses four stepwise binary logistic regression models. The first model in both tables tests the association between race and ethnicity and age on the presence or absence of respiratory symptoms. The second model appends gender to the first model specification. The third model adds geographic region to the second model specification. The fourth model tests whether the presence or absence of respiratory symptoms significantly differs when considering the interaction between patient race and ethnicity, and patient home region. Odds ratios (ORs) are calculated to interpret the association between patient characteristics and presence or absence of respiratory symptoms. We used 0.05 to determine statistical significance and also included 95% confidence intervals for parameter estimates. STATA 16.1 is used for the statistical analyses in this study.

## Results

### Descriptive Statistics

From January to June 2019, a total of 542,762 EMS activations with patient contact were recorded for adults aged 50 and older. However, 2,662 observations (0.5%) were excluded due to missing data in the variables of interest. The final analytic sample for EMS activations with patient contact between January to June 2019 was 540,100. From January to June 2020, a total of 587,606 EMS activations with patient contact were recorded for adults aged 50 and older. However, 2,531 observations (0.4%) were excluded due to missing data. The final analytic sample for EMS activations with patient contact from January to June 2020 was 585,075.

Descriptive statistics of EMS activations with patient contact among adults aged 50 and older in this sample are included in Table 1. Between January and June 2019, the gender distribution among adults aged 50 and older in this sample was 52% women and 48% men. Among this sample, 47% identified as White, 28% as Other race and ethnicity, 11% as Latino, 9% as Black, and 4% as Asian. In the sample, 36% lived in Southern California, 26% in the Bay Area, 16% in Northern California, 15% in Central California, and 7% in Other regions. Approximately 12% of adults aged 50 and older in the sample experienced respiratory symptoms between January and June 2019.

**Table 1:** EMS activations with patient contact for adults aged 50 and over between January and June 2019 and January and June 2020.

	**2019 (** ***N*** **= 540,100)**		**2020 (** ***N*** **= 585,075)**	
	**Frequency**	**%**	**Frequency**	**%**
**Respiratory Symptoms**				
Yes	65,729	12.17	75,455	12.90
No	474,371	87.83	509,620	87.10
**Age**				
50–54	53,413	9.89	57,567	9.84
55–59	66,885	12.38	73,267	12.52
60–64	68,910	12.76	77,253	13.20
65–69	64,704	11.98	71,769	12.27
70–74	63,606	11.78	71,131	12.16
75–79	59,875	11.09	65,396	11.18
80–84	58,349	10.80	61,392	10.49
85–89	53,002	9.81	55,243	9.44
90–94	36,047	6.67	36,418	6.22
95–99	13,101	2.43	13,347	2.28
100+	2,208	0.41	2,292	0.39
**Gender**				
Women	282,158	52.24	296,641	50.70
Men	257,942	47.76	288,434	49.30
**Race/Ethnicity**				
Other	152,057	28.15	196,161	33.53
Latino	59,327	10.98	57,958	9.91
Black	48,938	9.06	48,880	8.35
Asian	24,235	4.49	24,018	4.11
White	255,543	47.31	258,058	44.11
**Patient Home Region**				
Other	37,638	6.97	32,077	5.48
Northern California	87,228	16.15	101,955	17.43
Bay Area	141,526	26.20	151,650	25.92
Central California	79,635	14.74	96,242	16.45
Southern California	194,073	35.93	203,151	34.72

*California Emergency Medical Services Authority (30)*.

During the early pandemic period, January to June 2020, 51 and 49% of EMS calls with patient contact in this sample were among women and men, respectively. Among adults aged 50 and older in this sample, 44% were White, 34% were Other race and ethnicity, 10% were Latino, 8% were Black, and 4% were Asian. In the sample, 35% lived in Southern California, 26% in the Bay Area, 17% in Northern California, 16% in Central California, and 6% in Other regions. Lastly, 13% of adults aged 50 and older in the sample experienced respiratory symptoms during the early pandemic period.

### Inferential Statistics

Table 2 shows that from January to June 2019, Whites had higher odds of having respiratory distress related EMS calls compared to Latinos across all four models (Model 1, OR = 1.16, 95% CI: [1.13, 1.19]; Model 2, OR = 1.16, 95% CI: [1.13, 1.19]; Model 3, OR = 1.19, 95% CI: [1.15, 1.22]; Model 4, OR = 1.24, 95% CI: [1.18, 1.29]). Asians also had higher odds of having respiratory distress related EMS calls compared to Latinos, although the findings were not statistically significant after including the race by region interaction (Model 1, OR = 1.12, 95% CI: [1.07, 1.17]; Model 2, OR = 1.12, 95% CI: [1.07, 1.17]; Model 3, OR = 1.18, 95% CI: [1.15, 1.22]). Blacks had the highest odds of having respiratory distress related EMS calls compared to Latinos across all four models (Model 1, OR = 1.48, 95% CI: [1.43, 1.54]; Model 2, OR = 1.48, 95% CI: [1.43, 1.54]; Model 3, OR = 1.54, 95% CI: [1.48, 1.60]; Model 4, OR = 1.48, 95% CI: [1.39, 1.57]). Thus, from January to June 2019, Latinos had lower odds of having respiratory distress related EMS calls compared to the other racial or ethnic groups studied. No association between age and respiratory distress related EMS calls was identified among adults aged 50 and older after controlling for all other covariates. Likewise, no statistically significant differences in respiratory distress related EMS calls were identified between women and men across all models when adjusting for all other covariates. Central California had higher odds of respiratory distress related EMS calls compared to Southern California (Model 3, OR = 1.15, 95% CI: [1.12, 1.18]; Model 4, OR = 1.20, 95% CI: [1.12, 1.27]). The Bay area (Model 3, OR = 0.90, 95% CI = [1.12, 1.18]; Model 4, OR = 0.85, 95% CI: [0.79, 0.91]) and Other region (Model 3, OR = 0.70, 95% CI = [0.68, 0.73]; Model 4, OR = 0.73, 95% CI: [0.65,0.82]) had lower odds of respiratory distress related EMS calls compared to Southern California There were statistically significant interactions between race/ethnicity and region.

**Table 2:** Logistic regressions models: respiratory distress related calls by predictor specifications among adults aged 50 and over between January and June 2019.

	**Model 1**	**Model 2**	**Model 3**	**Model 4**
**Race/Ethnicity (Reference = Latino)**				
Other	1.01	1.01	1.03	1.00
Black	1.48^***^	1.48^***^	1.54^***^	1.48^***^
Asian	1.12^***^	1.12^***^	1.18^***^	1.09
White	1.16^***^	1.16^***^	1.19^***^	1.24^***^
**Age**	1.01^***^	1.00^***^	1.01^***^	1.01^***^
**Gender (Reference = Men)**				
Women		1.01	1.00	1.00
**Region (Reference = Southern California)**				
Other Region			0.70^***^	0.73^***^
Northern California			1.00	1.00
Bay Area			0.90^***^	0.85^***^
Central California			1.15^***^	1.20^***^
**Interaction: Race/Ethnicity and Region (Reference= Latino and**				
**Southern California)**				
White × Other Region				1.00
White × Northern California				0.96
White × Bay Area				0.97
White × Central California				0.92^*^
Black × Other Region				0.96
Black × Northern California				0.95
Black × Bay Area				1.17^**^
Black × Central California				1.01
Asian × Other Region				1.25
Asian × Northern California				1.10
Asian × Bay Area				1.14
Asian × Central California				1.08
Other × Other Region				0.86^*^
Other × Northern California				1.08
Other × Bay Area				1.29^***^
Other × Central California				0.93
**Wald test (** ***p*** **-value)**				0.000
**Constant**	0.07^***^	0.07^***^	0.07^***^	0.07^***^
**Observations**	540,100	540,100	540,100	540,100
**AIC**	398782.60	398784.20	398105.50	397965.50
**BIC**	398849.80	398862.60	398228.70	398267.90

*** *p < 0.001*,

** *p < 0.01*,

* *p < 0.05*.

Table 3 shows that from January to June 2020, the results are different compared to the same time period in the previous year. During the early pandemic period, Whites had lower odds of having respiratory distress related calls compared to Latinos across all four models (Model 1, OR = 0.91, 95% CI: [0.88, 0.93]; Model 2, OR = 0.91, 95% CI: [0.88, 0.93]; Model 3, OR = 0.92, 95% CI: [0.90, 0.95]; Model 4, OR = 0.89, 95% CI: [0.85, 0.93]). This finding differed from the previous year, which had higher odds of respiratory distress related calls among Whites compared to Latinos. The only racial group with statistically significant higher odds of respiratory distress related calls during the early pandemic period, across all models, were Blacks compared to Latinos (Model 1, OR = 1.19, 95% CI: [1.15, 1.23]; Model 2, OR = 1.19, 95% CI: [1.15, 1.23]; Model 3, OR = 1.22, 95% CI: [1.18, 1.26]; Model 4, OR = 1.10, 95% CI: [1.03, 1.16]). Results show no association between age and respiratory distress related EMS calls. No statistically significant differences in respiratory distress related EMS calls were identified between women and men after adjusting for all other covariates (Model 3, OR = 0.98, 95% CI: [0.97, 1.00]; Model 4, OR = 0.98, 95% CI: [0.97, 1.00]). As in the previous year, Central California also had marginally higher odds of respiratory distress related EMS calls compared to Southern California (Model 3, OR = 1.07, 95% CI: [1.05, 1.10]). Central California, however, had marginally lower odds of respiratory distress related EMS calls compared to Southern California after including a race by region interaction (Model 4, OR = 0.88, 95% CI: [0.83, 0.95]). All other regions had significantly lower odds relative to Southern California. Interactions between race/ethnicity and region show statistically significant results.

**Table 3:** Logistic regressions models: respiratory distress related calls by predictor specifications among adults aged 50 and over between January and June 2020.

	**Model 1**	**Model 2**	**Model 3**	**Model 4**
**Race/Ethnicity (Reference = Latino)**				
Other	0.90^***^	0.90^***^	0.87^***^	0.76^***^
Black	1.19^***^	1.19^***^	1.22^***^	1.10^**^
Asian	1.00	1.00	1.04	1.06
White	0.91^***^	0.91^***^	0.92^***^	0.89^***^
**Age**	1.00^***^	1.00^***^	1.00^***^	1.00^***^
**Gender (Reference = Men)**				
Women		0.99	0.98^*^	0.98^*^
**Region (Reference = Southern California)**				
Other Region			0.63^***^	0.69^***^
Northern California			0.93^***^	0.75^***^
Bay Area			0.88^***^	0.75^***^
Central California			1.07^***^	0.88^***^
**Interaction: Race/Ethnicity and Region (Reference = Latino and**				
**Southern California)**				
White × Other Region				0.90
White × Northern California				1.19^**^
White × Bay Area				1.06
White × Central California				1.14^**^
Black × Other Region				0.88
Black × Northern California				1.15^*^
Black × Bay Area				1.29^***^
Black × Central California				1.15^*^
Asian × Other Region				0.83
Asian × Northern California				1.05
Asian × Bay Area				1.06
Asian × Central California				1.09
Other × Other Region				0.87^*^
Other × Northern California				1.37^***^
Other × Bay Area				1.35^***^
Other × Central California				1.35^***^
**Wald test (** ***p*** **-value)**				0.000
**Constant**	0.12^***^	0.12^***^	0.13^***^	0.14^***^
**Observations**	585,075	585,075	585,075	585,075
**AIC**	449325.40	449326.30	448534.70	448347.20
**BIC**	449393.10	449405.20	448658.80	448651.70

*** *p < 0.001*,

** *p < 0.01*,

* *p < 0.05*.

## Discussion

Respiratory distress related EMS calls increased during the early pandemic period (31). Our study adds to the existing literature by examining respiratory distress related EMS calls by race and ethnicity during the early pandemic period, January to June 2020, compared to the same time period in 2019. Between January and June 2019, Latinos had lower odds of respiratory distress related EMS calls compared to Blacks, Asians, and Whites. This finding may be explained by lower-than-average access and use of health care services among Latinos (32). Further, Latino adults aged 50 and older with respiratory distress symptoms were less likely to use EMS perhaps due to general distrust in emergency services, financial concerns, immigration status, and patient-provider language discordance (13, 20, 22). On the contrary, between January and June 2020, Latinos had higher odds of respiratory distress related EMS calls compared to Whites. This finding may be indicative of the disproportionate effects of COVID-19 among Latino adults aged 50 and older.

It is also important to consider other reasons why respiratory distress related EMS calls increased among Latinos from January to June 2020. Future studies should consider possible changes in the availability of EMS resources in the regions under study, other possible exposures that patients may have experienced that contributed to respiratory distress, and policies that were passed during the early pandemic period that influenced patient decisions to call 9-1-1. For example, policies that reduced or waived the cost of EMS may have influenced patient behavior, particularly individuals from low socio-economic backgrounds, by addressing financial concerns. Media attention during the early pandemic period may have also contributed to increases in EMS use by spreading awareness on the symptoms of respiratory distress, whether or not they were related to COVID-19. Another possibility is that fear during the COVID-19 pandemic elevated the general population's threshold prior to seeking emergency services. By waiting longer to seek medical care, the use of emergency services became an even more important sector in the healthcare system during the COVID-19 pandemic, particularly for disproportionately impacted populations, including Latinos.

The statistically significant race/ethnicity and region interaction for both years suggests that there are regional and county-level differences in respiratory distress related EMS calls that need to be considered, although this analysis may be more appropriate when all 33 LEMSAs submit their data to CEMSIS. As discussed, Los Angeles County contributes a large part of Southern California's population. EMS data for Los Angeles County, however, are not currently submitted to CEMSIS.

Another important finding of our study was the higher odds of respiratory distress related EMS calls among Blacks compared to Latinos. This difference may allude to the impacts of structural racism, early health deterioration, and an accumulation of disadvantage over the lifetime experienced by Blacks in California (2, 33). The high use of EMS among Blacks has been documented even before the COVID-19 pandemic (34, 35). Access to affordable health insurance, quality healthcare services, and a prevention focused approach are proposed changes to address the health disparities and inequities that disproportionately affect racial and ethnic minority populations, including Latino and Black populations (34, 35).

## Limitations

The current study has some limitations. The first limitation is the exclusion of Los Angeles County from the CEMSIS dataset, the single state-wide database for EMS in California. The inclusion of Los Angeles County, where Latinos constitute 48% of the population, could strengthen the findings reported in this paper (36). Previous research shows that counties with a higher percentage of Latinos have higher numbers of confirmed COVID-19 cases (37). Among symptomatic COVID-19 cases, respiratory distress is one of the most common symptoms (38). Thus, after including Los Angeles County, the odds of respiratory distress related calls may be even higher among Latinos compared to Whites. The second limitation is the voluntary submission of EMS data by the LEMSAs and the lack of consistency on how often the data are reported. For instance, LEMSAs may report data weekly, monthly, or yearly. The LEMSA's different schedules to submit EMS data may result in a lag period for some of the data, which may also affect the findings reported in this study. The third limitation is the recording of gender as a binary construct in CEMSIS. The fourth limitation is the use of only EMS calls with patient contact in the analysis, which does not provide information on patients who were not on-scene. The fifth limitation is related to the collection of patient sociodemographic data. As described in the data analysis section, the CEMSIS patient race and ethnicity variable includes approximately 100 different categories. For the purposes of this study, individuals were coded as Latino, Black, Asian, White, and Other race and ethnicity if the patient's race was mixed, unknown, or not applicable. As a result, approximately 28 and 34% of individuals in 2019 and 2020, respectively, were classified as Other race and ethnicity. The high percentage in the Other race and ethnicity category may introduce some bias into the study. Future research should consider patient home county, or region as a proxy for race and ethnicity. Our study shows higher odds of respiratory distress related EMS calls in Central California compared to Southern California in some of the models. Central California is a region with one of the highest proportions of Latinos, particularly of low socioeconomic status and marginalized identities, such as undocumented migrant farm workers. Another limitation is the absence of other important sociodemographic variables from the dataset, such as immigration status, English language proficiency, and place of birth.

## Conclusion

Our study identifies racial and ethnic differences in EMS use in both the pre-COVID and early pandemic periods. Between January and June 2019, Latino adults aged 50 and older were less likely to report respiratory distress related EMS calls compared to Whites, Blacks, and Asians. However, during the early pandemic period, Latino adults aged 50 and older were more likely to report respiratory distress related EMS calls compared to Whites. We also found statistically significant race/ethnicity and region interactions. Understanding EMS health disparities is important to inform policies that create a more equitable prehospital care system that is responsive to an increasingly ethno-racially heterogeneous population of middle aged and older adults.

## Data Availability Statement

Publicly available datasets were analyzed in this study. The dataset may be requested here: https://emsa.ca.gov/cemsis/.

## Author Contributions

EM, HB-S, and AVB contributed to conception and design of the study. EM performed the statistical analysis. EM wrote the first draft of the manuscript. All authors contributed to manuscript revision, read and approved the submitted version.

## Conflict of Interest

The authors declare that the research was conducted in the absence of any commercial or financial relationships that could be construed as a potential conflict of interest.

## Publisher's Note

All claims expressed in this article are solely those of the authors and do not necessarily represent those of their affiliated organizations, or those of the publisher, the editors and the reviewers. Any product that may be evaluated in this article, or claim that may be made by its manufacturer, is not guaranteed or endorsed by the publisher.

## Appendix A

**Table A1:** Logistic regressions models: respiratory distress related calls by predictor specifications among adults aged 50 and over between January and June 2019.

	**Model 1**	**Model 2**	**Model 3**	**Model 4**
**Race/Ethnicity (Reference = Latino)**				
Other	1.00	1.00	1.03	1.01
Black	1.47^***^	1.47^***^	1.53^***^	1.47^***^
Asian	1.11^***^	1.11^***^	1.17^***^	1.08
White	1.15^***^	1.15^***^	1.17^***^	1.23^***^
**Age (Reference = 50–54 years)**				
55–59	1.25^***^	1.25^***^	1.24^***^	1.24^***^
60–64	1.49^***^	1.49^***^	1.48^***^	1.48^***^
65–69	1.62^***^	1.62^***^	1.61^***^	1.61^***^
70–74	1.71^***^	1.70^***^	1.69^***^	1.69^***^
75–79	1.74^***^	1.74^***^	1.722^***^	1.72^***^
80–84	1.63^***^	1.63^***^	1.61^***^	1.61^***^
85–89	1.53^***^	1.52^***^	1.51^***^	1.51^***^
90–94	1.55^***^	1.54^***^	1.54^***^	1.54^***^
95–99	1.67^***^	1.67^***^	1.67^***^	1.67^***^
100+	1.48^***^	1.48^***^	1.48^***^	1.49^***^
**Gender (Reference = Men)**				
Women		1.01	1.01	1.00
**Region (Reference= Southern California)**				
Other Region			0.71^***^	0.73^***^
Northern California			1.00	1.00
Bay Area			0.91^***^	0.86^***^
Central California			1.15^***^	1.20^***^
**Interaction: Race/Ethnicity and Region (Reference= Latino and**				
**Southern California)**				
White × Other Region				1.00
White × Northern California				0.95
White × Bay Area				0.96
White × Central California				0.93^*^
Black × Other Region				0.97
Black × Northern California				0.94
Black × Bay Area				1.15^**^
Black × Central California				1.01
Asian × Other Region				1.25
Asian × Northern California				1.10
Asian × Bay Area				1.14
Asian × Central California				1.09
Other × Other Region				0.86^*^
Other × Northern California				1.05
Other × Bay Area				1.27^***^
Other × Central California				0.92^*^
**Wald test (** ***p*** **-value)**				0.000
**Constant**	0.08^***^	0.08^***^	0.08^***^	0.08^***^
**Observations**	540,100	540,100	540,100	540,100
**AIC**	398034.40	398034.50	397388.80	397257.80
**BIC**	398202.40	398213.70	397612.80	397661.00

*California Emergency Medical Services Authority (30)*.

*** *p < 0.001*,

** *p < 0.01*,

* *p < 0.05*.

**Table A2:** Logistic regressions models: respiratory distress related calls by predictor specifications among adults aged 50 and over between January and June 2020.

	**Model 1**	**Model 2**	**Model 3**	**Model 4**
**Race/ethnicity (reference = Latino)**				
Other	0.90^***^	0.90^***^	0.87^***^	0.76^***^
Black	1.18^***^	1.18^***^	1.21^***^	1.09^**^
Asian	0.99	0.99	1.04^**^	1.05
White	0.90^***^	0.90^***^	0.91^***^	0.88^***^
**Age (reference = 50–54 years)**				
55–59	1.23^***^	1.23^***^	1.22^***^	1.22^***^
60–64	1.39^***^	1.39^***^	1.38^***^	1.38^***^
65–69	1.53^***^	1.53^***^	1.51^***^	1.51^***^
70–74	1.51^***^	1.51^***^	1.49^***^	1.49^***^
75–79	1.54^***^	1.54^***^	1.51^***^	1.51^***^
80–84	1.40^***^	1.40^***^	1.38^***^	1.39^***^
85–89	1.30^***^	1.30^***^	1.29^***^	1.29^***^
90–94	1.26^***^	1.26^***^	1.25^***^	1.26^***^
95–99	1.31^***^	1.31^***^	1.30^***^	1.31^***^
100+	1.41^***^	1.41^***^	1.41^***^	1.42^***^
**Gender (reference = Men)**				
Women		1.00	0.99	0.99
**Region (reference = Southern California)**				
Other Region			0.64^***^	0.70^***^
Northern California			0.92^***^	0.76^***^
Bay Area			0.88^***^	0.76^***^
Central California			1.07^***^	0.89^***^
**Interaction: Race/Ethnicity and Region (Reference = Latino and**				
**Southern California)**				
White × Other Region				0.90
White × Northern California				1.18^**^
White × Bay Area				1.06
White × Central California				1.14^**^
Black × Other Region				0.89
Black × Northern California				1.14^*^
Black × Bay Area				1.28^***^
Black × Central California				1.14^*^
Asian × Other Region				0.83
Asian × Northern California				1.06
Asian × Bay Area				1.06
Asian × Central California				1.09
Other × Other Region				0.87^*^
Other × Northern California				1.34^***^
Other × Bay Area				1.33^***^
Other × Central California				1.34^***^
**Wald test (** ***p*** **-value)**				0.000
**Constant**	0.12^***^	0.12^***^	0.13^***^	0.13^***^
**Observations**	585,075	585,075	585,075	585,075
**AIC**	448494.60	448496.50	447748.10	447577.50
**BIC**	448663.80	448677.00	447973.60	447983.50

*California Emergency Medical Services Authority (30)*.

*** *p < 0.001*,

** *p < 0.01*,

* *p < 0.05*.
